# 1-RAAP: An Efficient 1-Round Anonymous Authentication Protocol for Wireless Body Area Networks

**DOI:** 10.3390/s16050728

**Published:** 2016-05-19

**Authors:** Jingwei Liu, Lihuan Zhang, Rong Sun

**Affiliations:** State Key Laboratory of Integrated Services Networks, Xidian University, No.2, South Taibai Road, Xi’an, Shaanxi 710071, China; zhanglihuan678@163.com (L.Z.); rsun@mail.xidian.edu.cn (R.S.)

**Keywords:** wireless body area networks, security, sensors, anonymity, authentication protocol, 1-RAAP

## Abstract

Thanks to the rapid technological convergence of wireless communications, medical sensors and cloud computing, Wireless Body Area Networks (WBANs) have emerged as a novel networking paradigm enabling ubiquitous Internet services, allowing people to receive medical care, monitor health status in real-time, analyze sports data and even enjoy online entertainment remotely. However, because of the mobility and openness of wireless communications, WBANs are inevitably exposed to a large set of potential attacks, significantly undermining their utility and impeding their widespread deployment. To prevent attackers from threatening legitimate WBAN users or abusing WBAN services, an efficient and secure authentication protocol termed 1-Round Anonymous Authentication Protocol (1-RAAP) is proposed in this paper. In particular, 1-RAAP preserves anonymity, mutual authentication, non-repudiation and some other desirable security properties, while only requiring users to perform several low cost computational operations. More importantly, 1-RAAP is provably secure thanks to its design basis, which is resistant to the anonymous in the random oracle model. To validate the computational efficiency of 1-RAAP, a set of comprehensive comparative studies between 1-RAAP and other authentication protocols is conducted, and the results clearly show that 1-RAAP achieves the best performance in terms of computational overhead.

## 1. Introduction

Since the concept of Wireless Body Area Network (WBAN) was proposed in [1], it has drawn considerable attention from both academia and industry. The WBAN technology can be utilized in several applications such as physiological and medical monitoring, human computer interaction, and education, as well as entertainment. The technology provides a convenient environment to support and monitor the daily lives and medical conditions of patients without any restrictions. WBAN is a kind of short distance communication network consisting of various kinds of sensors. The sensors, which are attached to or implanted into the human body, could be used to collect and transmit important physiological signals (such as the temperature, the blood glucose, the blood pressure, *etc.*), human activities or action signals as well as information about the environment around a human’s body.

Despite the past non-trivial efforts, the WBAN concept still needs increasing research attention because of the openness of the wireless environment. In particular, the leakage of privacy is the major concern of potential users and must been taken into account. Due to its unique characteristics, such as open medium channel, signal noise, mobile terminals, *etc.*, WBANs encounter many security challenges in their practical applications. For example, in medical applications, authorized patients should share medical services under the circumstance that they just send the necessary information to the medical institution and the remaining private information such as their name must be kept unknown, so how to establish a suitable security mechanism to protect the privacy and security of transmitted and stored data which is vital to medical diagnosis and treatment represents an extraordinary imperative. In this paper, a 1-round anonymous authentication protocol is proposed to meet this requirement.

### 1.1. Related Work

In the authentication protocol based on Public Key Infrastructure (PKI), Certificate Authority (CA) in traditional Public Key Cryptography (PKC) [2,3] is required to issue and maintain a pool of certificates for the clients after verifying the validation of the clients, which results inevitably in the awkward certificate management problem. Various authentication schemes based on elliptic curve cryptosystem (ECC) have been proposed as alternatives [4,5,6,7,8], which have better performance thanks to the smaller key size in ECC [9]. For example, 160-bit ECC achieves the same security level as 1024-bit RSA. However, ECC-based ones also require a certification authority (CA) to maintain a pool of certificates for users’ public keys.

To overcome the drawbacks caused by the public key certificate, identity-based cryptosystem (IBC) was introduced by Shamir in 1984 and has rapidly developed after Boneh and Franklin’s first security-provable identity-based (ID-based) encryption using pairings. In IBC systems such as those described in [10,11,12,13,14,15,16,17,18,19,20], a client uses his or her identifier as a public key, while the corresponding private key is generated by a trusted Key Generation Center (KGC). IBC implements authentication without the storage, transmission and verification of public key certificates. However, for user’s private key depends entirely on KGC, so key escrow security becomes an inevitable challenge.

To solve the aforementioned problem, Al-Riyami *et al.* proposed certificateless public key cryptography (CL-PKC) [21] in 2003. The KGC will only process a partial private key for users according to the master key and user’s identity, and then the users combine it with a secret value selected by themselves to get their complete private key. So far, many CL-PKC-based schemes have been proposed in recent years [22,23,24,25,26,27,28,29,30,31]. In [30], the authors proposed a pair of efficient and light-weight certificateless authentication protocols to enable remote WBAN users to anonymously enjoy healthcare services, in which any opponents (even the application server) have no privileges to disclose the real identities of users. In [31], the authors proposed a remote anonymous authentication protocol with revocability for extra-body communication in WBANs. However, these schemes involved large amounts of computation.

Due to WBANs’ restrained resources, such as their poor computation capability, low-power and memory space, the existing protocols do not fit the WBAN very well. In this paper, we propose a 1-round anonymous authentication protocol, named 1-RAAP, to enhance the security characteristics, reduce the length of signature, provide higher computational efficiency, and take the energy consumption in account.

### 1.2. The Main Results

With the stated objectives in mind, we propose a novel authentication protocol for WBANs. The main contributions of this paper include:

- A 1-round anonymous authentication protocol—1-RAAP—is proposed. This scheme achieves mutual authentication, non-reputation, anonymity and session key establishment and is validated to be more secure and efficient than the existing ones.
- Relying on a test and an analysis of the performance of the proposed protocol, the results show that our scheme is better suited to WBANs.

### 1.3. Paper Outline

The rest of the paper is organized as follows: in Section 2, we provide a review of the definitions for groups equipped with bilinear maps and several complexity assumptions. The proposed 1-RAAP and its security properties analysis are thoroughly presented in Section 3. Computational efficiency and the performance evaluation are given in Section 4 followed by the conclusions presented in Section 5.

## 2. Preliminaries

### 2.1. Bilinear Pairings

**Definition 1.**  *Bilinear Pairings map. A bilinear pairing is defined as a map* $e:G_{1}\times G_{1}=G_{2}$, *where* $G_{1}$ *is a cyclic additive group generated by* P, *whose order is a prime* q, *and* $G_{2}$ *is a cyclic multiplicative group of the same order. We assume that the discrete logarithm problems (DLP) in both* $G_{1}$ *and* $G_{2}$ *are hard. Bilinear pairings have the following properties:*

- Bilinearity
  *Let* $P,Q\in G_{1}$, *random number* $a,b\in Z_{q}^{*}$, *then* $e\left(aP,bQ\right)=e{\left(P,Q\right)}^{ab}$;
- Non-degeneracy
  *There exists* $P,Q\in G_{1}$, *such that* $e\left(P,Q\right)\neq I_{G_{2}}$, *where* $I_{G_{2}}$ *denotes the identity element of group* $G_{2}$;
- Computability
  *There is an efficient algorithm to compute* e(P,Q), *for all* $P,Q\in G_{1}$;

### 2.2. Complexity Assumptions

**Definition 2.**  *Decisional Diffie-Hellman (DDH) Problem.* $G_{1}$ *is a cyclic additive group of prime order* q, P *is the generator of* $G_{1}$, *for any* $a,b,c\in Z_{q}^{*}$, *given an instance* 〈aP,bP,cP〉, *it is difficult to decide whether* abP=cP.

**Definition 3.**  *Divisible Computational Diffie-Hellman (DCDH) Problem.* $G_{1}$ *is a cyclic additive group of the prime order* q, P *is the generator of* $G_{1}$, *for* $a\in Z_{q}^{*}$, *give an instance* 〈aP,bP〉, *it is difficult* *to compute* $\frac{b}{a}P$ *and* abP.

## 3. 1-Round Anonymous Authentication Protocol for WBANs

In this section, 1-RAAP is specifically presented.

### 3.1. Definitions and Protocol Description

**Figure 1:**
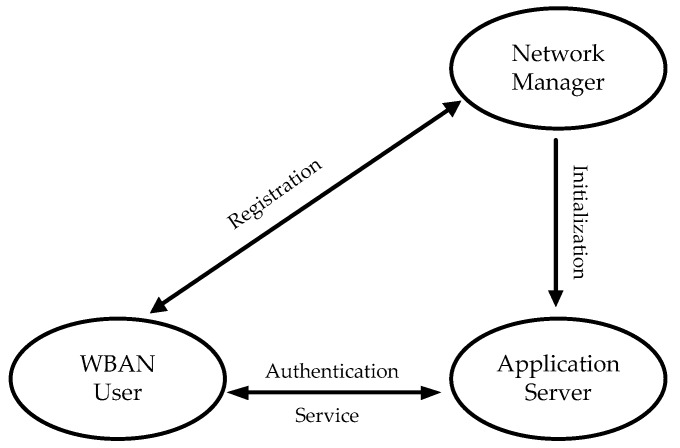
The participants of 1-RAAP.

The proposed anonymous authentication protocol contains three entities, shown as Figure 1.

- Network Manager (NM): it serves as a user management server in WBANs application scenarios;
- WBAN User: it refers to the user who uses certain WBAN terminals or applications such as a PDA, smart phone, biosensor or medical device to regularly access various medical services that are offered by Application Server.
- Application Server (AS): it provides corresponding services to authorized users, including patient monitoring, physician consult, and so on. It can be a hospital, clinic, physician and even a weather forecast station.

The user first registers to be a legitimate user of the system before enjoying the service, and then sends request to the server to acquire the related information. Upon receiving the request, the server first checks its database to verify the legitimacy of the user, and then provide related services to the valid user.

1-RAAP implements mutual authentication between the WBAN user and the application server, and guarantees that the user can gain access to the services anonymously. In other words, the server provides service to the authenticated user without knowing who he really is. Despite knowing the user’s account index, the server has no idea about who is asking for service. In addition, the user cannot deny that he has ever logged in the system to use the service because no one without the private key can successfully authenticate. The proposed 1-RAAP meets the features of WBAN, so the server can implement efficient and secure relevant services.

#### 3.1.1. Initialization

System is set up by NM, generating keys and establishing an enrollment system. In this step, NM determines its public/private key pair $\langle s_{NM},PK_{NM}\rangle$, where $PK_{NM}=s_{NM}P$, and publicizes the system parameters $\left\{l,G_{1},G_{2},q,P,H,PK_{NM}\right\}$, in which l represents the security parameters. We suppose that AS also has a long-term key pair $\langle s_{AS},PK_{AS}\rangle$, where $PK_{AS}=s_{AS}P$. Each user generates a pair of public or private key $\langle s_{U},PK_{U}\rangle$, here $PK_{U}=s_{U}P$. $\left(G_{1},+\right)$ and $\left(G_{2},•\right)$ are a cyclic additive group and cyclic multiplicative group of the same prime order q, H is a secure hash function, $H:{\left\{0,1\right\}}^{*}\times G_{1}\to Z_{q}^{*}$.

#### 3.1.2. Registration

**Figure 2:**
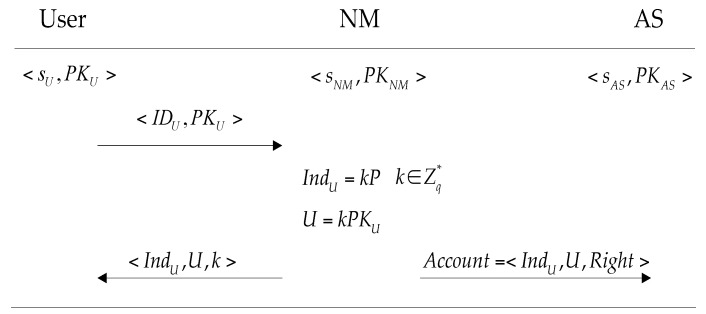
The registration of 1-RAAP.

Each user must execute this stage (shown as Figure 2) before accessing the services. The user sends his identity $ID_{U}$ and public key $PK_{U}$ to the network manager, and the network manager chooses $k\in Z_{q}^{*}$ randomly, then computes a user index $Ind_{U}=kP$ and $U=kPK_{U}$ for the user, the network manager simultaneously issues $\langle Ind_{U},U,k\rangle$ to the user and $Account=\langle Ind_{U},U,Right\rangle$ to the server. Here, Right indicates auxiliary information such as service type and prescriptive period. This phase should be carried out under the security channel. We believe that NM is reliable, which is a prerequisite and the basis of trust. So the *k* will not be leaked to others.

#### 3.1.3. Authentication

**Figure 3:**
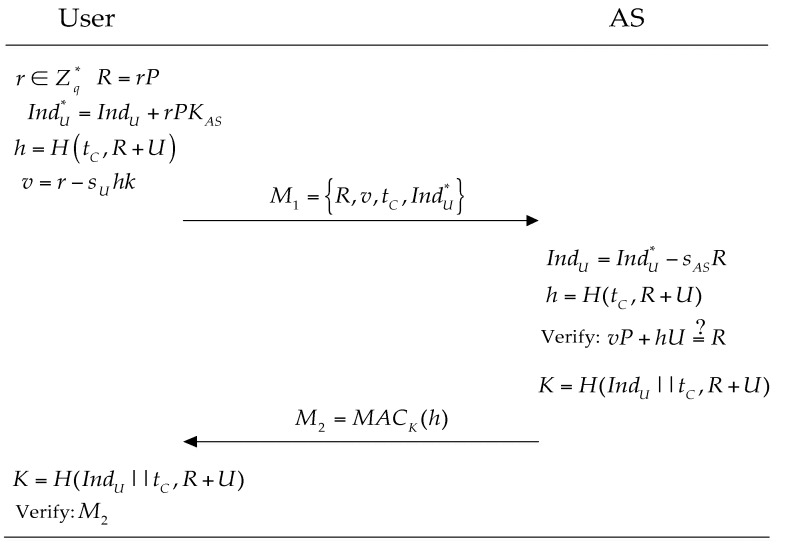
The authentication of 1-RAAP.

The WBAN user should perform the following steps to prove him/herself to AS when she/he needs to obtain relevant information, shown as Figure 3. Otherwise, the protocol terminates immediately.

- Select $r\in Z_{q}^{*}$ randomly and compute R=rP and $Ind_{U}^{*}=Ind_{U}+rPK_{AS}$.
- Pick up the current time $t_{c}$ and compute $h=H\left(t_{c},R+U\right)$.
- Compute $v=r-s_{U}hk$
- Send a service request message $M_{1}=\left\{R,v,t_{c},Ind_{U}^{*}\right\}$ to the AS.

On receiving the service request message $M_{1}$, AS first checks the validity of the time stamp $t_{c}$ and then computes $Ind_{U}=Ind_{U}^{*}-s_{AS}R$ and searches the database with the user’s index $Ind_{U}$ and verifies whether the equation $vP+hU\;\underline{\underline{?}}\text{​ }R$ holds, here $h=H\left(t_{c},R+U\right)$. If the equation holds, we consider that the user is legitimate. The AS will perform the following steps:

- Compute the session key: $key=H\left(Ind_{U}\|t_{c},R+U\right)$.
- Compute $M_{2}=MAC_{key}\left(h\right)$.

AS sends $M_{2}$ to the user. Upon receiving the response message $M_{2}$, the user computes the session key. Then, the user checks the integrity of the message authentication code by the session key. If the result is negative, the user quits the current session.

We then carefully examined the operational efficiency of 1-RAAP and compared it to those of the existing schemes. Table 1 summarizes the results, in which PCM means the point multiplication in $G_{1}$, EXC means the exponentiation computation in $G_{2}$, BP means the bilinear pairings computation.

Table 1 shows that our scheme involves two point multiplication operations in $G_{1}$ and two hash operations on the client side, and three point multiplication operations in $G_{1}$, two hash operations on the server side. Both sides do not have exponentiation computation in $G_{2}$ and bilinear pairings. Generally, the pairing operation is several times more complex than the scalar multiplication in $G_{1}$. Thus, the number of pairing operations is a key performance metric. It is easily observed that our scheme is significantly simplified and can obtain higher efficiency as a whole.

### 3.2. Security Analysis

In this subsection, we give a comparison between our scheme and the other existing schemes. It demonstrates that our scheme provides higher security level. We will analyze the seven security characteristics of the 1-RAAP authentication protocol provided specifically.

#### 3.2.1. Anonymity

When a WBAN user registers to the system, network manager randomly chooses $k\in Z_{q}^{*}$ for the user to generate an index $Ind_{U}$ and signature U. Then the user authenticates to the WBAN service network and application server (AS) who provides service to the authenticated user. However, AS does not know who the authenticated user really is in this process. The advantages of the anonymous requests for services are to avoid the leakage of the private information and increase the flexibility of authentication.

**Definition 4.**  *An authentication scheme achieves anonymity, if for any probabilistic polynomial time adversary* $A_{ℐ}$, $\mathrm{Pr}[\prod \leftarrow O_{\prod }(ID)]$; $ID^{\prime }\leftarrow A_{ℐ}:ID^{\prime }=ID$ *is negligible.*

Anonymity means that an adversary $A_{ℐ}$ cannot obtain the real identity of any WBAN client based on the existing communication. Now we formalize a game: when an oracle $O_{\prod }\left(ID\right)$ outputs a session message P of a legitimate user with the identity ID $A_{ℐ}$ tries to reveal id with the help of AS.

**Theorem 1. (Anonymity)**  *The 1-RAAP meets anonymity, assuming the hardness of DCDH described in Section 2*.

**Proof.**  Suppose adversary $A_{ℐ}$ is a probabilistic polynomial time Turing machine who tries to reveal an anonymous user’s real ID corresponding to any existing session massage with non-negligible probability after getting enough experience. Simulator C has strong ability to imitate any state of whole communication environment and share all information with AS, who may be a malicious AS.

When C receives a DCDH instance $\langle Ind_{U},U\rangle =\langle kP,ks_{U}P\rangle$. Its goal is to compute $PK_{U}=s_{U}P$ to find the corresponding ID. C gives the parameters $\left\{l,G_{1},{\text{G}}_{2},q,P,H,PK_{NM}\right\}$ and $\langle Ind_{U},U\rangle$ to $A_{ℐ}$. It attempts to simulate the challenger by simulating all the oracles to obtain the ID of client U In particular, $A_{ℐ}$ can query as follows:

- H-Queries: $A_{ℐ}$ can query the random oracle H at any time. C simulates the random oracle by keeping a list of couples $\langle {⊥}_{i},h_{i}\rangle$ that is called L_H_, where ${⊥}_{i}$ is a couple of $\langle x_{i},{ϒ}_{i}\rangle$, where $x_{i}\in {\left\{0,1\right\}}^{*}$, and ${ϒ}_{i}\in G_{1}$. When the oracle is queried with an input ⊥, C responds as follows:
  1. If the query ⊥ is already in the item of $\langle ⊥,h_{i}\rangle$ in L_H_, C outputs $h_{i}$.
  2. Otherwise, C selects a random $h\in Z_{q}^{*}$, outputs h and adds 〈⊥,h〉 to L_H_.
- Initial-Queries: C simulates the initial massage sent by any WBAN client U with $\langle Ind_{U},U\rangle$ and $t_{c}$. C answers the query as follows:
  1. C picks up a random h, $v\in Z_{q}^{*}$ where h is not equal to any existing output of H oracle.
  2. C computes R=vP+hU. If $⊥=\langle t_{c},R+U\rangle$ equals to any previous input of H oracle, then it returns to step 1.
  3. C adds 〈⊥,h〉 to L_H_.
  4. C computes $Ind_{U}^{*}=Ind_{U}+s_{AS}R$ and outputs $\left(R,v,t_{c},Ind_{U}^{*}\right)$ as the initial message $M_{1}$ sent from client U.
- Respond-Queries: C simulates the respond massage sent by AS with $\left(R,v,t_{c},Ind_{U}^{*}\right)$. C answers the query as follows:
  1. C computes $h=H\left(t_{c},R+U\right)$ and R=vP+hU.
  2. C computes $key=H\left(Ind_{U}\|t_{c},R+U\right)$.
  3. C outputs $MAC_{key}\left(h\right)$ as the response message $M_{2}$ sent from AS.

Thus, the initial message can be generated without knowing the private key $s_{U}$ of user U. All oracles, simulated by C, has high quality; $A_{ℐ}$ is fully satisfied with the all queries’ answers. It can fully exert its ability.

Eventually, given an input of $\left(R,v,t_{c},Ind_{U}^{*}\right)$, adversary $A_{ℐ}$, with non-negligible probability, outputs a legal public key $PK_{U}$ of client U and reveals the real ID from $PK_{U}$. Here, $\left(R,v,t_{c},Ind_{U}^{*}\right)$ is not any output of Initial-Queries. C then successfully solves $\langle Ind_{U},U,PK_{U}\rangle$ = $\langle kP,ks_{U}P,s_{U}P\rangle$. It obviously contradicts the hardness of the DCDH problem.

**Definition 5.**  *An authentication scheme achieves unlinkability, if for any probabilistic polynomial time adversary* $A_{ℐℐ}$ *in the above UL Game,* $Adv_{A_{ℐℐ}}=\left|\mathrm{Pr}[\langle \prod_{1},\prod_{2}\rangle \leftarrow O_{\prod }(b),b^{\prime }\leftarrow A_{ℐℐ}:b^{\prime }=b]-\frac{1}{2}\right|$ *is negligible.*

**Theorem 2. (Unlinkability)**  *The security-enhanced anonymous authentication protocol achieves unlinkability, assuming the hardness of DDHP described in Section 2*.

Unlinkability [32,33,34,35] means that an adversary $A_{ℐℐ}$ cannot distinguish WBAN clients based on their communication. This means that the all session messages generated by clients should not leak any information to $A_{ℐℐ}$that allows $A_{ℐℐ}$to trace them. Now, similar to [35], we formalize UL Game: when an oracle $O_{\prod }\left(b\right)$ for b∈(0,1) outputs two session messages $\left(\prod_{1},\prod_{2}\right)$ with two identical (b=0) or two different (b=1) legitimate clients, $A_{ℐℐ}$ guesses b∈(0,1) with the help of NM.

**Proof.**  Suppose adversary $A_{ℐℐ}$ is a probabilistic polynomial time Turing machine whose input consists of public data. It can represent two identical or two different WBAN client from two given session massages with non-negligible probability after getting enough experience. Simulator C has a strong ability to imitate any state of the whole communication environment and share all information with NM, who maybe a malicious NM. When C receives a DDH instance (aP,bP,Q) its goal is to decide if Q=abP. C gives the parameters $\left\{l,G_{1},G_{2},q,P,e,H,h,Q_{PKG}\right\}$ to $A_{ℐℐ}$. It attempts to simulate the challenger by simulating all the oracles. In particular, $A_{ℐℐ}$can query as follows:

– **H-Queries**: Same as in Theorem 1.
– **Initial-Queries**: Same as in Theorem 1.
– **Respond-Queries**: Same as in Theorem 1.

Thus, the initial message can be generated without knowing the partial private key $s_{U}$ of client U. All oracles, simulated by C, have high quality; $A_{ℐℐ}$ is fully satisfied with the all queries’ answers. It can fully exert its ability.

Eventually, given two sessions of $\left(R_{1},v_{1},t_{c1},Ind_{U1}^{*}\right)$ and $\left(R_{2},v_{2},t_{c2},Ind_{U2}^{*}\right)$, adversary $A_{ℐℐ}$, with non-negligible probability, outputs “0” or “1” (Note: “0” means $I_{1}=I_{2}$ and “1” means $I_{1}\neq I_{2}$). Here, $\left(R_{1},v_{1},t_{c1},Ind_{U1}^{*}\right)$ and $\left(R_{2},v_{2},t_{c2},Ind_{U2}^{*}\right)$ are not any output of **Initial Queries**. Without knowing $s_{AS}$, abP can solve a DDH instance:

$$\left(aP=|s_{as}P=PK_{as},bP=R_{2}-R_{1},Q=Ind_{U1}^{*}-Ind_{U2}^{*}\right)$$

with the help of $A_{ℐℐ}$, because:

$$\begin{array}{l} Ind_{U1}\;\underline{\underline{?}}\;Ind_{U2}\\ \Leftrightarrow \;Ind_{U1}^{*}-s_{as}R_{1}\;\underline{\underline{?}}\;Ind_{U2}^{*}-s_{as}R_{2}\\ \Leftrightarrow \;s_{as}\left(R_{2}-R_{1}\right)\;\underline{\underline{?}}\;Ind_{U1}^{*}-Ind_{U2}^{*}\\ \Leftrightarrow \;abP\;\underline{\underline{?}}\;Q \end{array}$$

This obviously contradicts the hardness of the DDHP problem.

#### 3.2.2. Mutual Authentication

1-RAAP realizes the mutual authentication between the user and the server. On receiving the request and the signature *U* from the user, the server searches its database with the account index *Ind_U_* to ensure the existence of the user and then verifies the authenticity of the user by using the user’s public key. If all processes hold, the server sends the message authentication code to the user. Then, the user first verifies whether the message authentication code is equal to the value he computed by himself. If so, the user verifies the signature to determine the validity of the server.

#### 3.2.3. Non-Repudiation

For R is generated by the user, no one can forge it without the information on the user’s private key, so the user cannot deny that he has ever requested the services provided by the server.

#### 3.2.4. Session Key Establishment

The server and the user will negotiate a session key during authentication process. Only the user and the server know the session key.

#### 3.2.5. Immunity of Key Escrow

This protocol is based on the scheme described previously that can solve the inherent key escrow problem in general anonymous authentication protocol. This property can be obtained directly from Theorem 1.

#### 3.2.6. Unforgeability

The information of user and AS cannot be forged in our protocol. The first condition, if there is an adversary who try to pretend to be a legal user, he cannot get the value *k*. Even he forges a fake key, AS will calculate the *Ind_U_* and compare it with the one from the fake user. If they are not equal, the identity of the adversary will be exposed. The second condition, if there is an adversary who want to pretend to be the AS, he does not know *S_AS_*, and can’t calculate the *Ind_U_*. From the above, we can conclude that our protocol has unforgeability.

#### 3.2.7. Forward Security

The proposed 1-RAAP can provide the forward security property under the DCDH assumption. Suppose that the private key of the AS or the private key of a User were corrupted after establishing a session key shared by the AS and the user. Let *a* and *b* be the ephemeral key used by the AS and the User during the establishment of the shared session key respectively. Obviously, in order to compute *abP* in the shared session key, the adversary who has obtained the full private key must solve the DCDH problem in *G*_1_ without the knowledge of either *a* or *b*. Therefore, our protocol provides the property of forward security.

### 3.3. Security Features Comparison

We compare the security features of our protocol with other existing authentication protocols described in [4,6,10,11,12,18,19,30,31], with the results shown in Table 2.

Compared with the other schemes, the proposed 1-RAAP is more secure and provides thorough privacy protection. To sum up, 1-RAAP realizes the mutual authentication between the user and the server, the user can obtain the corresponding service under the condition that the user’s key information will not be leaked.

## 4. Performance Evaluation

We are particularly concerned about the computational complexity and energy consumption of 1-RAAP. To validate that, we set up simulations and compare 1-RAAP with several typical existing schemes. We first analyze the message size which is related to energy consumption on message propagation. Then, a detailed analysis on computational time is provided, along with discussions about energy consumption on both message transmission and computation. 

### 4.1. Message Size

Due to the significant effect of the message size on the energy consumption, we start by analyzing the message size of the following schemes.

- The Certificate-Based Authentication Scheme in [2]: the total message size of the scheme is equal to $\left|M|+|tt|+|SIG|+|Cert_{U_{ID}}\right|$; here |*| denotes the size of “*” in bytes. The minimum size of the $\left|Cert_{U_{ID}}\right|$ is 86 bytes according to the method mentioned in [36]. According to [37], we know *SIG* is |q| bytes. Then we assume message size of *M* is 20 bytes, the time stamp *tt* is 2 bytes, and |q| is 20 bytes, so the message size of the certificate-based authentication scheme is 128 bytes.
- A mutual authentication and key exchange scheme in [6]: the total message size of the scheme is equal to $\left|ID_{i}|+|SIG|+|U|+|t_{1c}|+|t_{2c}|+|Auth\right|$. Similarly, $ID_{i}$ is the address of 2 bytes, SIG is |q| bytes, U is an element of $G_{1}$ of the order |q|, $t_{1c}$ and $t_{2c}$ are time stamps of 2 bytes repectively, Auth is a hash value of 20 bytes given by SHA-1. Then we can calculate the message size is 66 bytes.
- Identity-Based Anonymous Remote Authentication scheme in [10]: the total message size of the scheme is equal to $\left|ID_{sp}|+|R^{\prime }|+|SIG|+|t_{c}|+|MAC\right|$. Using the same assumption, $ID_{sp}$ is the address of 2 bytes, $R^{\prime }$ is an element of $G_{1}$ of the order |q|, SIG is |q| bytes, $t_{c}$ is a time stamp of 2 bytes, MAC is 20 bytes given by SHA-1, so the message size is 64 bytes.
- The *ID*-Based Authentication Scheme in [11]: the total message size of the scheme is equal to $\left|ID_{U}|+|\text{x}|+|SIG|+|Z|+|t_{u}\right|$. Here $ID_{U}$ denotes the user’s address of 2 bytes, |x| and *t_u_* are elements of $G_{1}$ and of $Z_{q}$, respectively, with the same order |q|, and *Z* is a hash value which should be 20 bytes given by SHA-1, similarly the SIG is |q| bytes, so the message size is 82 bytes.
- An efficient remote user authentication and key agreement protocol in [19]: the total message size of the scheme is equal to $\left|ID_{C}|+|U|+|r|+|Auth|+|R_{c}|+|V\right|$. As above, $ID_{C}$ is the address of 2 bytes, U, $R_{c}$ and V are elements of $G_{1}$ of the order |q|, r is the element of $Z_{q}$ of the order |q|, Auth is a hash value of 20 bytes given by SHA-1. The message size is 102 bytes.
- Certificateless Remote Anonymous Authentication scheme in [30]: the total message size of the scheme is equal to $\left|v|+|U|+|t_{c}|+|T\prime |+|I\prime |+|MAC\right|$. Using the same assumption, $t_{c}$is the address of 2 bytes, U, T′ and I′ are elements of $G_{1}$ of the order |q|, v is the element of $Z_{q}$ of the order |q|, MAC is a hash value of 20 bytes given by SHA-1. The message size is 102 bytes.
- Revocable and Scalable Certificateless Remote Anonymity Authentication scheme in [31]: the total message size of the scheme is equal to $\left|C_{0}|+|C_{1}|+|C_{2}|+|C_{3}|+|R_{B}|+|MAC\right|$. Using the same assumption, $C_{0}$, $C_{1}$, $C_{2}$, $C_{3}$ and $R_{B}$ are elements of $Z_{q}$ of the order |q|, MAC is a hash value of 20 bytes given by SHA-1. The message size is 120 bytes.
- The proposed 1-RAAP authentication protocol: its total message size is equal to $\left|Ind_{U}^{*}|+|R|+|t_{c}|+|v|+|MAC\right|$. Assuming that everything else is the same as above, $Ind_{U}^{*}$, R are the elements of the $G_{1}$ of the order |q|, v is the element of $Z_{q}$ of the order |q|, $t_{c}$ is a time stamp of 2 bytes, and the MAC is 20 bytes given by SHA-1. Thus we obtain that our scheme’s size is 82 bytes.

**Figure 4:**
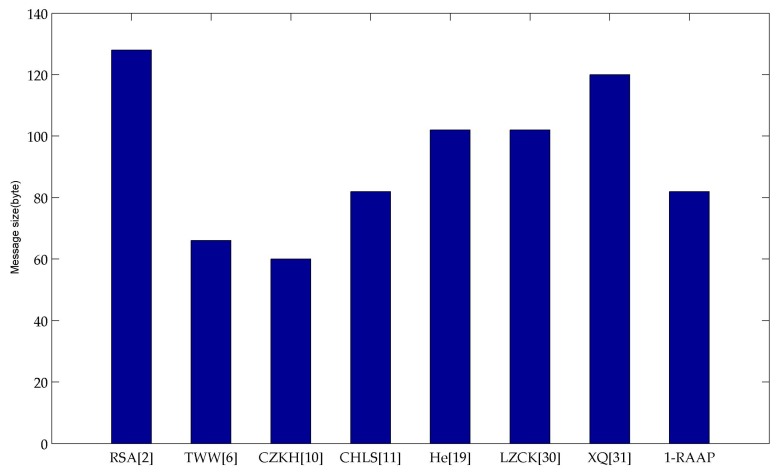
Message size comparison of different schemes.

Figure 4 shows the message sizes of different schemes. From them, we can arrive at the following conclusions:

- Firstly, the certificate-based authentication scheme in [2] has the maximum message size due to the existence of the certification.
- Secondly, we can further see that the message size of the Identity-Based Anonymous Remote Authentication scheme in [10] is the minimum, but according to the scheme in [30], the message size of the ID-based scheme increases with the increased value of |q|. In our comparison, we assume the |q| is 20 bytes, so it is clear this scheme will not have the minimum message size when |q| increases.
- Finally, neither the maximum nor minimum one in message size, our scheme does not seem to have the obvious advantages over others. However, by the following analysis, our scheme shows a better trade off.

### 4.2. Computational Time

From Section 4.1, it is clear that certificate-based scheme with relatively greater message size is not quite suitable to WBAN, so in the remaining sections, the features of other selected schemes will be quantified except the certificate-based scheme. Now, we analyze the computational efficiency of these schemes.

#### 4.2.1. Simulation Environment Setup

In this subsection, we set up the simulation hardware environment to measure the computational time of these selected schemes. The simulation environment of AS is Windows XP OS on an Inter(R)Pentium IV 3.0GHz processor and 512 MB memory. The hardware environment of a typical mobile WBAN client, such as a PDA, has a low-power high-performance 32-bit Inter(R) PXA270 624MHz processor [38] and 128MB memory running Windows CE 5.2OS. In addition, we set the pair operation is defined over a supersingular elliptic curve $y^{2}=x^{3}+x$. The run time of cryptographic primitives on the AS is obtained by experiment and that on the client terminal is estimated using the method in [37]. The simulations will run several times and the results are averaged to compensate for the randomness. Moreover, we set the message authentication code to 160 bits.

#### 4.2.2. Simulation Results

Noting that the computational overhead mainly results from the cryptographic operations, for the sake of simplicity we thus use the computational time consumed on different cryptographic operations as an approximation of the computational overhead. Table 3 lists the run time of several cryptographic operations. In the selected schemes, the computation overhead is mainly due to the cryptographic operations of exponentiation in $Z_{q}^{*}$, multiplication in $G_{1}$ and pairing. 

**Figure 5:**
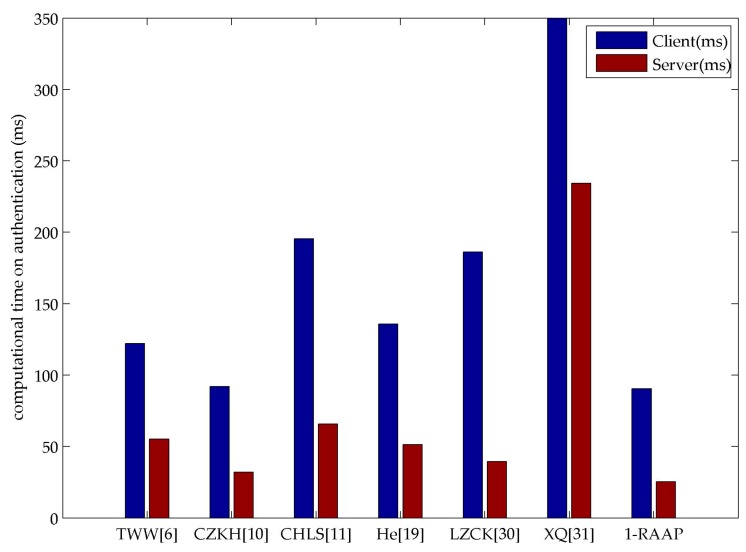
Different schemes’ computational time on authentication.

Given the cryptographic operations and their corresponding time consumption, we can calculate the computational time on authentication process of the selected schemes (shown as Figure 5).

By comparing with the other schemes in different phases, it is clear that our scheme performs better. We note that the server takes the most time in 1-RAAP, but what cannot be neglected is the phenomenon that the server terminal spends most time on the initialization phase that will only run once at the beginning of the system’s setup, 1-RAAP costs the least in total authentication process time after being initialized. It is obviously more efficient than others. It proves that 1-RAAP successfully transfers the calculation burden to the server whose computing ability is relatively stronger. Also, it saves the energy consumption in the user terminal. These merits make 1-RAAP very suitable in the WBAN scenario. 

### 4.3. Energy Consumption

In this subsection, the evaluation of the energy consumption has two aspects: first, we consider the effect of message propagation on the energy consumption; second, we take the computation overhead into account. Eventually, we make an in-depth analysis of the pros and cons of each scheme.

We use the same method in [39] to evaluate the energy consumption due to the transmission of the messages with different size. As is reported, a Chipcon CC1000 radio used in Crossbow MICA2DOT motes consumes 28.6 uJ and 59.2 uJ to receive and transmit one byte, respectively, at an effective data rate of 12.4 kb/s. Moreover, we assume a packet size of 41 bytes, 32 bytes for the payload and 9 bytes for the header. The header, ensuing an 8-byte preamble, consists of source, destination, length, packet ID, CRC, and a control byte. Thus receiving one 41-bytes packet (in addition to the 8-byte preamble) costs 49 × 28.6 = 1.40 mJ, and the corresponding transmission costs 49 × 59.2 = 2.90 mJ. Knowing this, we can calculate the total energy overhead of every scheme as follows:

(1) The Certificate-Based Authentication Scheme in [2]: From Section 4.1, we know the message size of this scheme is 128 bytes and then we take the following steps to calculate the energy overhead.
  - Divide the message into four packets in total, all of them are 41 bytes.
  - The bytes to be transmitted are: 41 × 4 + 8 × 4 = 196 bytes, and the relevant energy overhead is 196 × 59.2 = 11.60 mJ.
  - The bytes to be received are: 196 bytes, and the related energy consumption is 196 × 28.6 = 5.61 mJ.
(2) The ID-Based Authentication Scheme in [11]: The message size of this scheme is 82 bytes. We do the same steps to obtain the energy overhead.
  - Divide the message into three packets in total, among which two of them are 41 bytes, and one is 27 bytes.
  - The bytes to be transmitted are: 41 × 2 + 27 × 1 + 8 × 3 = 133 bytes, and the relevant energy overhead is 133 × 59.2 = 7.87 mJ.
  - The bytes to be received are: 133 bytes, and the related energy consumption is 133 × 28.6 = 3.80 mJ.
(3) A mutual authentication and key exchange scheme in [6]: The message size of this scheme is 66 bytes. The energy overhead is calculated using the following steps:
  - Divide the message into three packets in total, among which two of them are 41 bytes, and one is 11 bytes.
  - The bytes to be transmitted are: 41 × 2 + 11 × 1 + 8 × 3 = 117 bytes, and the relevant energy overhead is 117 × 59.2 = 6.93 mJ.
  - The bytes to be received are: 117 bytes, and the related energy consumption is 117 × 28.6 = 3.35 mJ.
(4) Identity-Based Anonymous Remote Authentication scheme in [10]: The message size of this scheme is 64 bytes. We do the same steps to obtain the energy overhead:
  - Divide the message into two packets in total, both of them are 41 bytes.
  - The bytes to be transmitted are: 41 × 2 + 8 × 2 = 98 bytes, and the relevant energy overhead is 98 × 59.2 = 5.80 mJ.
  - The bytes to be received are: 98 bytes, and the related energy consumption is 98 × 28.6 = 2.80 mJ.
(5) An efficient remote user authentication and key agreement protocol in [19]: The message size of this scheme is 102 bytes. Then we take the following steps to calculate the energy overhead:
  - Divide the message into four packets in total, among which three of them are 41 bytes, and one is 15 bytes.
  - The bytes to be transmitted are: 41 × 3 + 15 × 1 + 8 × 4 = 170 bytes, and the relevant energy overhead is 170 × 59.2 = 10.06 mJ.
  - The bytes to be received are: 170 bytes, and the related energy consumption is 170 × 28.6 = 4.86 mJ.
(6) Certificateless Remote Anonymous Authentication scheme in [30]: The message size of this scheme is 170 bytes. We do the same steps to get the energy overhead:
  - Divide the message into four packets in total, among which 3 of them are 41 bytes, and one is 15 bytes.
  - The bytes to be transmitted are: 41 × 3 + 15 × 1 + 8 × 4 = 170 bytes, and the relevant energy overhead is 170 × 59.2 = 10.06 mJ.
  - The bytes to be received are: 166 bytes, and the related energy consumption is 170 × 28.6 = 4.86 mJ.
(7) Revocable and Scalable Certificateless Remote Anonymity Authentication scheme in [31]: The message size of this scheme is 188 bytes. The energy overhead can be calculated as follows:
  - Divide the message into four packets in total, among which 3 of them are 41 bytes, and one is 33 bytes.
  - The bytes to be transmitted are: 41 × 3 + 33 × 1 + 8 × 4 = 188 bytes, and the relevant energy over head is 188 × 59.2 = 11.13 mJ.
  - The bytes to be received are: 166 bytes, and the related energy consumption is 188 × 28.6 = 5.38 mJ.
(8) 1-RAAP: From Section 3, we know the message size of 1-RAAP is 82 bytes, so the energy overhead is calculated as follows.
  - Divide the message into four packets in total, among which two of them are 41 bytes, and one is 27 bytes.
  - The bytes to be transmitted are: 41 × 2 + 27 × 1 + 8 × 3 = 133 bytes, and the relevant energy overhead is 133 × 59.2 = 7.87 mJ.
  - The bytes to be received are: 166 bytes, the related energy consumption is 3.80 mJ.

**Figure 6:**
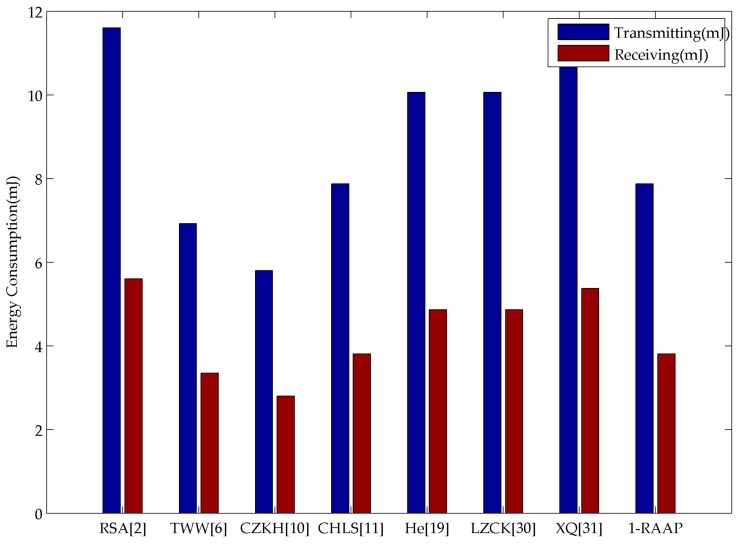
Energy consumption on message transmission.

Figure 6 shows that 1-RAAP offers a relatively lower energy message propagation overhead as compared to the others, while the scheme in [31] consumes the most energy.

In order to facilitate comparisons, we sum up the performance evaluation comparison between the different authentication protocols in Table 4.

The results in Table 4 demonstrate that the proposed 1-RAAP generally outperforms the others and offers a better tradeoff between the security properties and performance. We would like to design a protocol with better trade-off between computational overhead and energy consumption, so that the computational complexity of the authentication protocols can be decreased as a whole. These makes it more suitable for wireless body area networks.

## 5. Conclusions

A secure 1-round anonymous authentication protocol for WBAN—1-RAAP—is proposed in this paper. All the user operations involved in the scheme require a very small amount of calculation. Complex computation is transferred to a server with relatively higher computing ability. The security properties of mutual authentication, non-reputation, anonymity, and session key establishment allow users to securely access the services at any time. Furthermore, the analysis of energy consumption demonstrates our scheme has higher efficiency. To sum up, the proposed 1-RAAP authentication scheme can achieve a better performance compared with the current schemes, and provides communication services efficiently and securely for WBAN users.

## Figures and Tables

**Table 1:** Comparison of computational complexity.

The Schemes	Client						Server		
	BP	EXC	PCM	Hash		BP	EXC	PCM	Hash
TWW [6]	0	0	3	2		2	0	1	3
CZKH [10]	0	0	2	2		0	1	1	2
CHLS [11]	0	1	3	3		2	1	1	3
He [19]	0	0	3	3		1	1	1	4
LZCK [30]	0	1	3	2		1	1	1	2
XQ [31]	1	12	1	7		8	4	0	6
1-RAAP	0	0	2	2		0	0	3	2

**Table 2:** Security feature comparison between different authentication protocols. “√” indicates that the property is satisfied.

Scheme	He [19]	DSGP [18]	GDS [4]	WT [12]	CZKH [10]	CHLS [11]	TWW [6]	LZCK [30]	XQ [31]	1-RAAP
Anonymity								√	√	√
Mutual Authentication	√			√	√	√	√	√	√	√
Session Key Establishment	√			√	√	√	√	√	√	√
Non-repudiation	√	√	√	√	√	√	√	√	√	√
Immunity of key escrow								√	√	√
Unforgeability								√		√
Forward Security							√		√	√

**Table 3:** Computational time consumed on different cryptographic operations.

Operations	Server (ms)	Client (ms)
Exponentiation in $Z_{q}^{*}$	13.21	63.51
Multiplication in $G_{1}$	6.38	30.67
Hash in $G_{1}$	3.14	14.62
Pairing	20.04	96.35

**Table 4:** The performance evaluation comparison between different authentication protocols.

Schemes	TWW [6]	CZKH [10]	CHLS [11]	He [19]	LZCK [30]	XQ [31]	1-RAAP
Message Size (byte)	66	64	82	102	102	120	82
Client’s Computational Time (ms)	122.08	92.06	195.52	135.87	186.19	990.05	90.58
Server’s Computational Time (ms)	55.08	32.08	65.67	51.34	39.63	233.44	25.42
Transmitting Energy Consumption (mJ)	6.93	5.80	7.87	10.06	10.06	11.13	7.87
Receiving Energy Consumption (mJ)	3.35	2.80	3.80	4.86	4.86	5.38	3.80
